# Facile Fabrication of Highly Perforated Hollow Metallic Cylinder with Changeable Micro-Orifices by Electroforming-Extrusion Molding Hybrid Process

**DOI:** 10.3390/mi11010070

**Published:** 2020-01-09

**Authors:** Junzhong Zhang, Pingmei Ming, Xinmin Zhang, Ge Qin, Liang Yan, Xiaokang Zhao, Xingshuai Zheng

**Affiliations:** Institute of Non-traditional Machining & Equipment, Henan Polytechnic University, Jiaozuo 454000, China; zhangjunzhong0520@163.com (J.Z.); qinge@hpu.edu.cn (G.Q.); yanliang@hpu.edu.cn (L.Y.); xchen@mail.nwpu.edu.cn (X.Z.);

**Keywords:** electroforming, perforated hollow metallic cylinder, changeable micro-orifice, extrusion molding, electrodeposition

## Abstract

A seamless thin-walled hollow metallic cylinder with array of micro-perforations is one of the key components for some products. Normally, these micro-perforations are formed by removing material from the given metallic hollow cylinder (pipe or tube) one by one or row by row. To efficiently and flexibly manufacture such a highly perforated hollow cylinder, this paper proposed a hybrid technique combining extrusion moulding process and electroforming process. In the hybrid technique, the extrusion moulding process was used to create polymer extrusion patterns on the outside surface of the given stainless steel (SS) pipe, and then the electroforming process was carried out using the SS pipe as the mandrel. The formation of the polymer extrusion patterns was simulated and extruding molding experiments were carried out to examine the feasibility of the various mandrels. Electroforming experiments were implemented to verify the achievement of the seamless perforated thin-walled hollow cylinder. It was found that five different types of polymer extrusion pattern were able to be obtained on the same extruding pipe just by adjusting some extruding conditions and parameters, and correspondingly four types of perforated hollow cylinder with different tapered orifices are produced after the electroforming process. The obtainable perforations are: perforation with double conic-orifices, perforation with hemispheric orifice and conic orifice, unidirectionally tapered perforation, and straight-walled perforation. The geometric profile of the extrusion patterns is highly dependent on the processing conditions and parameters. The proposed hybrid process represents a promising alternative process to fabricate seamless thin-walled perforated hollow metallic cylinder efficiently, flexibly, and with low cost.

## 1. Introduction

Thin-walled metallic articles and components featuring a variety of micro-perforations are frequently used for various industrial and consuming products, such as nickel rotary screen for textile printing, stencil for surface mounting technology (SMT), filter sieve, shaver foil, fine metal mask (FMM) for OLED, etc. [1,2,3,4,5]. Some of them are functionalized in the form of a seamless hollow cylinder like nickel rotary screen and filter sieve [6]. Normally, such a perforation cylinder is fabricated by the subtractive process, i.e., removing materials from the given blank metallic cylinder and the micro-perforations are formed one by one. These subtractive processes include mechanical drilling, punching, high energy beam machining (laser beam drilling, electron beam drilling, electrodischarge drilling, and electrochemical etching, etc. [7,8], but they are either less flexible, inefficient, or underqualified to meet the production demand. For example, laser beam drilling is good at forming circular tapered micro-perforations, but is very hard to make noncircular and/or high aspect ratio holes efficiently, and further, the laser beam-ablated surface is usually rough and covered with the recast layer. Electroforming, a typical additive metallic precision forming process, is a preferred process to form perforated precision micro-features on a large scale with good surface quality [9,10,11,12,13]. It is also facile to produce various geometric features. As a perforation-making process, electroforming is able to be used in two different manners: one is through the mask deposition manner in which metal is deposited into the active area formed by the thick-film photoresist patterns, and the other is the over-growth deposition manner in which metal is over-deposited surrounding the thin-film photoresist patterns. Lightly tapered perforated features are frequently produced with the through-mask deposition manner, and significantly tapered perforated features are formed with the over-growth deposition manner.

Until now, most of the perforated components and articles are formed on the planar conductive substrate by reversely duplicating the shape of photoresist patterns using the electroforming process. The photoresist patterns are normally prepared by the photolithographic process. Flat perforated thin plate products such as SMT stencil, lead frame, and ultraprecision nebulization plate are generally fabricated by a photolithography-electroforming hybrid process, which is very tedious and costly. However, the standard photolithographic process makes it extremely difficult to prepare the required photoresist patterns on the curved substrate, since thick photoresist film is not able to be uniformly coated on the curved surface, and homogeneous radiation of the exposure energy on such a surface is not easy be realized. Therefore, to manufacture curved perforated plates and hollow cylinders, the preparation of the photoresist patterns on the metallic substrate used as the electroforming mandrel needs to be significantly innovated. Herein, we present a typical example of electroforming nickel rotary screen. The nickel rotary screen which is a thin-walled hollow cylinder with a vast number of significantly tapered micro-perforations is manufactured mainly with the over-growth deposition manner. Preparation of the photoresist patterns on the cylinder metallic mandrel for electroforming the nickel rotary screen are distinct from the standard photolithographic process. Preparation involves a series of operation steps: shaping the cylinder, pre-treating the cylinder surface, depositing nickel coating, depositing copper coating, precision-turning and polishing the cylinder, forming cavities, depositing chrome coating, filling photoresist material into the cavities and over-growth depositing nickel, etc. [14,15,16]. However, the over-growth electrodeposition process used for the fabrication of the nickel rotary screen is not flexible to fulfill the demand of forming various orifices, because it can only produce the unidirectionally tapered perforations, but fails to fabricate other types of the perforations such as double conic orifices, hemispheric-conic double orifices, straight-wall orifices, etc. By contrast, the through-mask electrodeposition is capable to produce various perforated features including the highly tapered ones by inversely duplicating the shape of the mandrels as long as the mandrels can be fabricated, so the electroforming process via through-mask electrodeposition mechanism is significantly flexible in the industrial applications. 

This paper proposes a novel technique to fabricate seamless thin-walled hollow metallic cylinders with a vast number of micro-perforations by using the combination of the electroforming process and extrusion molding process. In the proposed technique, the seamless perforated hollow metallic cylinder is created by such a two-step process: form extrusion polymer patterns via an extrusion molding operation on the outside surface of a perforated stainless steel (SS) pipe, and subsequent electrodeposition of metal on the SS pipe. The proposition of this technique results from such a concept: the geometric shape of the extrusion polymer pattern during polymer extrusion molding can be flexibly changed just by altering the operating conditions. Correspondingly perforations with versatile geometric profile are expected to be obtained with the inversely duplicating mechanism after electroforming operations are implemented. In the following sections, feasibility of this hybrid process is investigated numerically and experimentally after the working principle is introduced.

## 2. Numerical Analysis

### 2.1. Over-Growth Electroforming Principle

**Figure 1:**
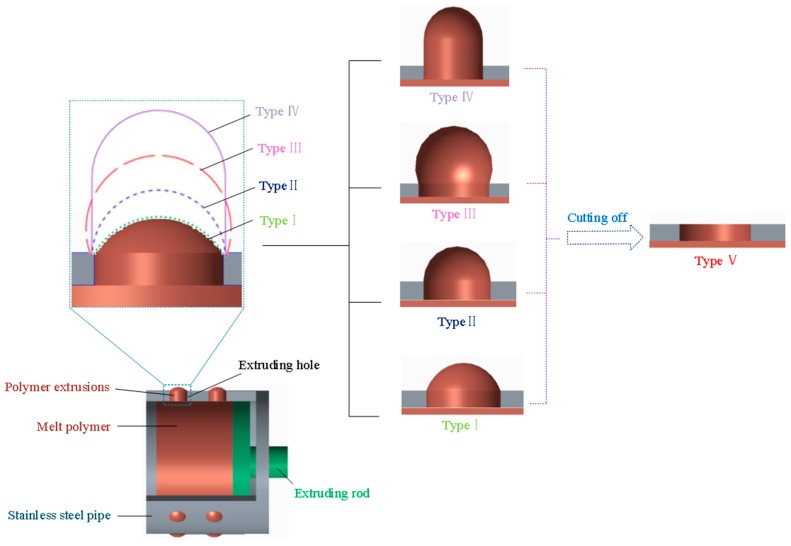
Schematic diagram of preparation of the versatile polymer patterns for electroforming mandrel by extrusion molding process.

As shown in Figure 1, five types of protruded polymer pattern are expected to be formed theoretically on the outside surface of the extruding SS pipe by the polymer extrusion molding. These solid polymer patterns can be in various shapes: gibbous shape (type I), domed shape (type II), spherical shape (type III), and cylindrical dome shape (type IV), depending on the primary operating conditions including extruding time, polymer temperature, solidification method and solidification time, etc. Correspondingly, at least four types of perforations are able to be achieved if using these polymer patterns as the mandrels of the through-mask electrodeposition process. Uniquely, another polymer pattern is able to be easily obtained only by cutting off the polymer extrusions described-above along the outside surface of the extruding pipe. The top surface of the obtained polymer pattern aligns with the outside surface of the extruding pipe. This type of polymer pattern is called type V pattern. With the type V pattern, significantly tapered perforations are expected to be produced via the over-growth electrodeposition electroforming process. Accordingly, it can be deduced that five types of perforated cylindrical electroforms will be obtained using the proposed hybrid technique.

**Figure 2:**
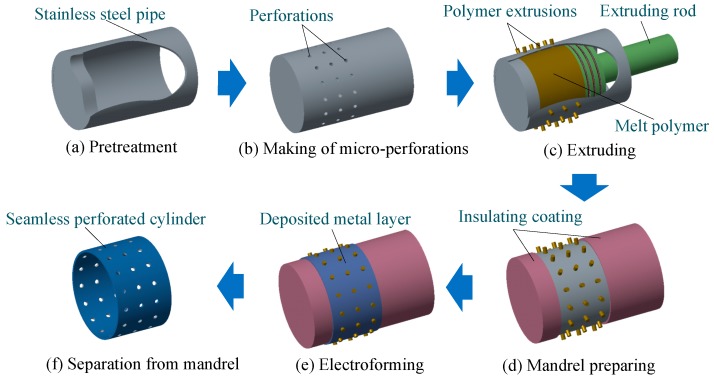
Main procedures of the hybrid technique for manufacture the seamless thin-walled perforated hollow metallic cylinder.

The main procedures of the described hybrid technique are illustrated in Figure 2. Firstly, thin-walled SS pipe is prepared prior to the fabrication of arrays of perforations on the cylinder SS pipe. Thirdly, extrusion polymer patterns are formed on the surface of the pipe by the extrusion molding process. Fourthly, metal is electroformed using the SS pipe with polymer patterns as the mandrel. Fifthly, the deposited electroformed article is separated from the pipe and then removed from the extrusion polymer patterns. In such a way, at least four types of thin-walled perforated hollow metallic cylinder are expected to be obtained by using the mandrel with the different polymer patterns.

### 2.2. Model Development and Numerical Solution

According to electrodeposition theories, geometric shape of the electroformed perforation is highly dependent on the geometric shape of the polymer pattern on the mandrel because electroforming is actually a duplication process. To better understand the feasibility of obtaining the desired feature, the formation of the polymer pattern during the polymer extrusion molding are simulated numerically.

**Figure 3:**
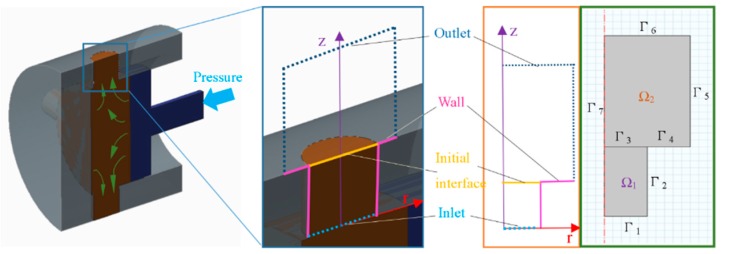
2D model for simulation of formation of the polymer pattern during extrusion molding.

For the sake of simplicity, a 2D model is established (shown in Figure 3) in which the Ω1 domain represents the melt polymer and Ω2 domain represents the air, while Γ_7_ is referred to as the axis of the model and Γ_3_ is referred to as the interface between the air and the melt polymer. Some assumptions are made as follows:

(1) the melt polymer is viewed as a kind of viscous fluid and is homogeneous in its material properties;
(2) temperature of the melt polymer keeps constant and is the same everywhere;
(3) neglecting the influence of gravity during polymer extruding-out;
(4) outlet pressure of the melt polymer is set to be zero;
(5) feeding rate of the extruding rod keeps constant;
(6) melt polymer is considered as an incompressible liquid.

To understand the shape evolution of the polymer extrusions, the level set method is adopted. The level set method, which was first proposed by Osher et al. (1988) [17], is widely used to calculate the intricate movement of interface. With the level set method, the shape of one phase is determined by the interface of the two contact phases. According to the research results of Lan W. et al. [17,18,19,20], merging and splitting of the interface can be well defined by using this method, which means the shape evolution of the interface with time can be numerically illustrated by the level set method during simulation. 

The final geometric shape of the extruded polymer pattern is, of course, determined by the ultimate shape of the two-phase interface between the solid polymer and the air, and the geometric shape of the extrusion pattern can be presented by simulation using the level set function (see Equation (1)) which has been proven very effective in solving two-phase interface problem [21]. Generally, the phase property is described by the specific value of the function Φ. For example, the phase is considered to be solid and gas respectively if Φ = 1 and Φ = 0, and it is regarded as the interface between the liquid (melt polymer) and the gas (air) when Φ = 0.5.

∂Φ/∂t + ∇⋅(Φu) + γ[(∇⋅(Φ(1 − Φ)·∇Φ/|∇Φ|)) − ε∇⋅∇Φ] = 0(1)

where u is the moving velocity of the interface, γ is the initial velocity of the interface, and ε is the thickness of the interface.

The density ρ and kinematic viscosity u of the interface are given as follows:

ρ = ρ_air_ + (ρ_polymer_ − ρ_air_)Φ (2)

μ = μ_air_ + (μ_polymer_ − μ_air_)Φ (3)

where ρ_polymer_ and ρ_air_ are the density of the air and the melt polymer respectively, while u_air_ and u_polymer_ are the kinematic viscosity of the air and the melt polymer respectively.

The Navier–Stokes equation and continuity equation are employed to describe mass and momentum transfer of the interface which are presented as follows:

ρ(∂u/∂t + u⋅∇u) − ∇⋅(μ(∇u + ∇u^T)) + ∇p = F_st_ (4)

(∇⋅u) = 0(5)

where p is pressure and F_st_ is the surface tension of the interface. The surface tension, F_st_, is determined by the following formula:

F_st_ = ∇⋅T (6)

T = σ(I − (nn^T))δ(7)

where I is the unit matrix, n is the interface normal, σ is the surface tension coefficient (N/m), δ is the delta function, which is nonzero only at the fluid interface. The estimation formula of the normal interface is presented as follows [22]:

δ = 6|Φ(1 − Φ)|.(8)

The boundary conditions are defined as follows:

(9) $$u_{\left(\mathrm{Inlet}\right)}{|\Gamma }_{1}\;=\;k\left(R-r\right)\left(r-R\right)$$

(10) $$u_{\left(\mathrm{wall}\right)}{|\Gamma }_{2,4}\;=\;0$$

(11) $$p_{\left(\mathrm{Outlet}\right)}{|\Gamma }_{5,6}\;=\;0.\;$$

where k is a constant which depends on the initial velocity of the melt polymer at the inlet, r is a variable, and R is the radius of the circular inlet.

Commercial COMSOL Multiphysics (5.2a version, COMSOL Inc., Stockholm, Sweden) is used to simulate the geometric shape of the extrusion pattern. The required initial values of some parameters used for the numerical analysis are listed in Table 1. To enhance simulation accuracy, the interface is finely meshed in an adaptive mode.

### 2.3. Analysis of Simulation Results

**Figure 4:**
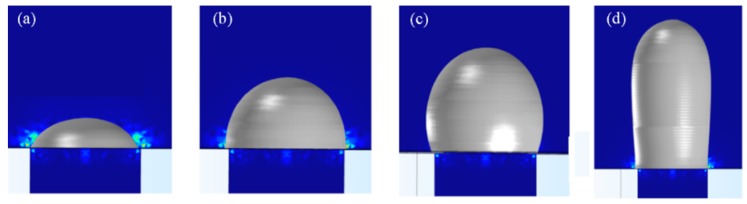
Simulated geometric profile of the polymer pattern formed by extrusion molding process with different operating condition. (**a**) Gibbous shape pattern; (**b**) Domed shape pattern; (**c**) Spherical shape pattern; (**d**) Cylindrical dome pattern.

Figure 4 shows the simulated geometric shape of the extrusion polymer pattern obtained under four different operating conditions. If the melt polymer is extruded out from the circular outlet with a constant velocity and in a very short time, the resultant geometric shape of the extrusion is a gibbous, i.e., type I (Figure 4a). When the extruding time is slightly extended with the other conditions for the type I being unchanged, dome-shaped extrusion pattern is obtained, as shown in Figure 4b (i.e., type II). However, when the melt polymer used is more viscous than those for the former two types of polymer pattern, and further a relatively longer extruding time is used, the achieved extrusion pattern is presented in a special shape, that is, an approximately spherical shape, as shown in Figure 4c (type III). This means that, besides the extruding time, the melt polymer, which is closely related to the viscosity of the melt polymer, is another critical factor determining the resultant geometric shape of the extrusion pattern. Surprisingly, when the extruding velocity is greatly enhanced and the viscosity of the melt polymer is reduced, cylindrically dome-shaped extrusion pattern is achieved which is shown in Figure 4d. 

In summary, as described above, four types of different extrusion polymer pattern are able to be obtained numerically only by changing some operating parameters during the polymer extrusion molding based on the same SS pipe. This verifies the feasibility of the proposed idea. In the following sections, the related verification experiments are depicted and their results are analyzed.

## 3. Experimentation

**Figure 5:**
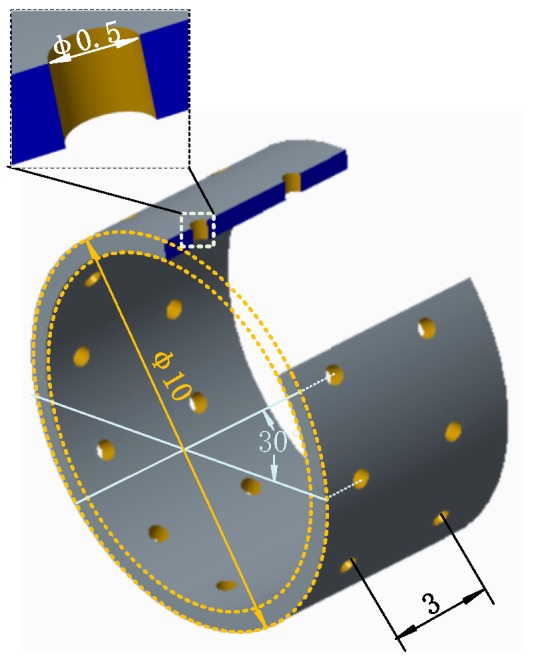
Geometric parameters of the perforated stainless steel pipe used as an extruding mold.

A stainless steel (SUS 304) pipe (outside diameter, 10 mm; inner diameter, 9 mm) with its one end being seamed was used as the extruding mold. Circular micro-perforations with straight wall were fabricated on the stainless steel pipe. The parameters of the perforated pipe used as the substrate for the electroforming are shown in Figure 5. Ethylene-vinyl acetate copolymer resin (PEVA or EVA) with a melt point of 130 °C was utilized as the extrusion polymer material. Main extrusion molding parameters and conditions are: heating temperature, 150–210 °C; feeding rate of the extruding rod, 0.5–1 mm/s. Cooling of the melt extrusion after finishing the extruding process was operated by keeping the extruded polymer in the distilled water at 5 °C for about 30 s.

To comprehensively evaluate the practicability of the proposed hybrid technique, the experiments using the electroforming mandrels with five types of polymer pattern shown in Figure 1 were implemented. Electrolyte compositions and process parameters used for Ni electrodeposition are listed in Table 2. A DC power supply was employed to the electrodeposition process and the applied current density was constantly 2 A/dm^2^. The electroforming time was 4 h. The polymer extrusions were removed chemically after the electroforming process. Topologies and morphologies of the polymer extrusions and the formed micro-perforations were examined by digital camera, scanning electron microscope (Merlin Compact, Carl Zeiss NTS GmbH, Oberkochen, German; JEOL JCM6000, Tokyo, Japan) and digital microscope (VHX-2000, KEYENCE, Osaka, Japan).

## 4. Results and Discussion

### 4.1. Achievement of the Seamless Hollow Cylinder with Double Conic-Tapered Perforations

**Figure 6:**
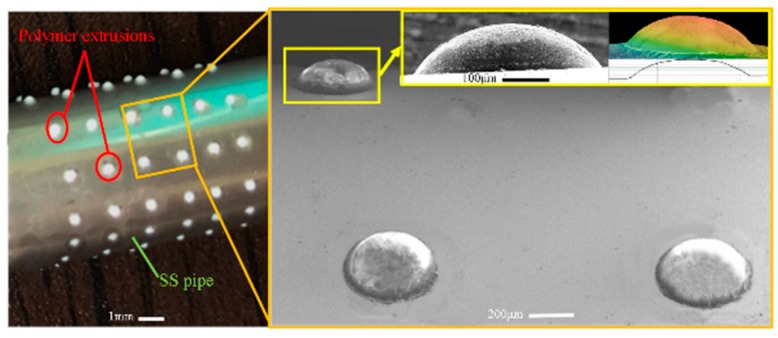
Electroforming mandrel with gibbous extrusion polymer patterns.

It was found experimentally that gibbous extrusion patterns can be formed under such conditions: heating temperature of the polymer, 210 °C; feed rate of the extruding rod, 0.5 mm/s; feed time, 1.5 s. As shown in Figure 6, the formed polymer extrusion pattern looks like the head of pushpin and is very smooth in its surface. The shape of the polymer pattern highly agrees with the simulated result.

**Figure 7:**
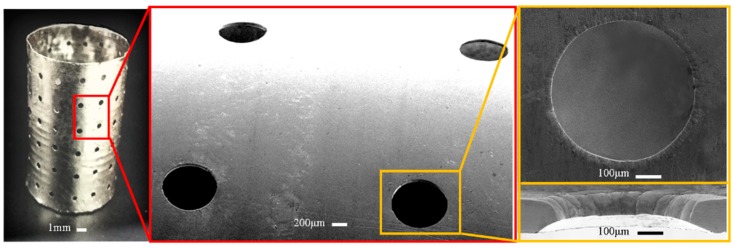
Electroformed seamless hollow cylinder with perforations with double conic-tapered orifices.

Figure 7 shows the electroformed seamless thin-walled hollow cylinder with perforations which are produced based on the polymer pattern of type I. It was found that the perforations with double conic-tapered orifices can be achieved as expected, and the two orifices have almost the same conic profile. There is slightly embosses around the orifice, and the film thickness is a little bit bigger than other zones regardless of the perforation type. This is caused by concentration of the current at the interfaces between the polymer extrusions and the deposit. However, the formation mechanism of the two orifices is not the same: one orifice is formed by duplicating the shape of extrusion polymer pattern via through-mask electrodeposition mechanism and the other is achieved by the over-growth electrodeposition process.

### 4.2. Achievement of the Seamless Hollow Cylinder with Perforations Having Hemisphere-Cone Double-Tapered Orifices

**Figure 8:**
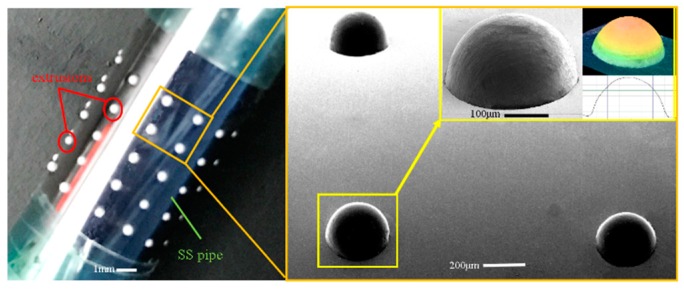
Electroforming mandrel with domed shape extrusion polymer pattern.

Compared with the former case described in the Section 4.1, the formation of the hemisphere-shaped polymer pattern (type II) is significantly complex. The main operating parameters and conditions for this type of polymer pattern are: feed rate of the extruding rod, 0.5 mm/s; stop feeding of the extruding rod as soon as the melt polymer was extruded out of the perforations of the SS pipe, and at the same time the SS pipe was immediately placed horizontally and simultaneously rotated at the speed of 20–30 rpm for 5–6 s before cooling. The above-described additional operations polymer occur as the melt is enabled to flow out and then to solidify naturally under the interactions of the gravity and the surface tension effects. Figure 8 illustrates the formed polymer extrusions (polymer mask) which show hemispherical geometry with very smooth surface. This indicates the real geometric profile of the mandrel agrees well with the simulated profile.

**Figure 9:**
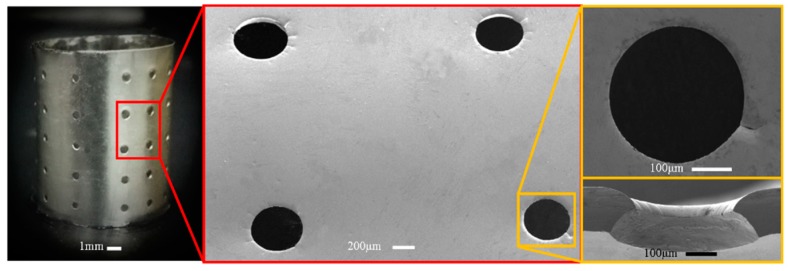
Electroformed seamless hollow cylinder with perforations with hemisphere-cone double-tapered orifices.

Figure 9 illustrates the electroformed perforated hollow cylinder based on the type II polymer mandrels with hemispherical geometry. It was found that the perforations with double-tapered orifices can also be obtained, but the geometric profiles of the two orifices are different in that one orifice is hemispherical and the other is conic. There is also slightly embosses around the orifice. Although the formation mechanisms of this kind of perforated hollow cylinder are very similar to those described in Section 4.1 based on the type I mandrels, the resulting geometric profile of the orifices varies. This is caused by the increase in the size of the extruded polymer patterns.

### 4.3. Achievement of the Seamless Hollow Cylinder with Unidirectionally Tapered Perforations

**Figure 10:**
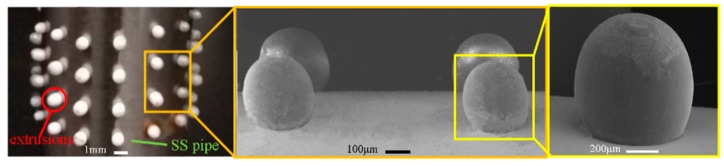
Electroforming mandrel with spherical extrusion polymer patterns.

**Figure 11:**
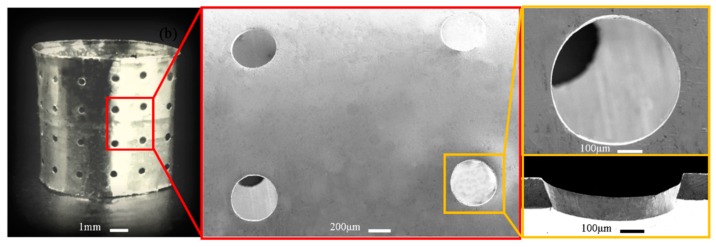
Electroformed seamless perforated hollow cylinder with unidirectionally tapered perforations.

**Figure 12:**
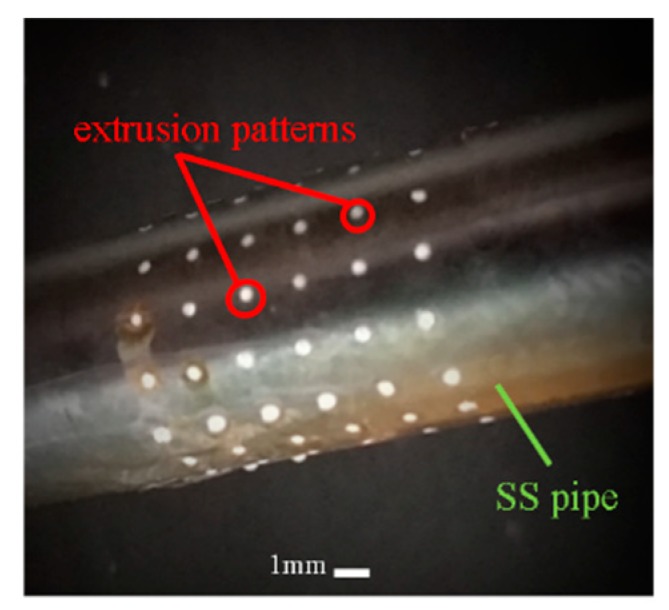
Electroforming mandrel with extrusion polymer patterns with their top surface aligning with the outside surface of the SS pipe.

As described previously, there are two mechanisms to control the formation of the electroformed features: reversely duplicating the shape of the mandrel and over-growth of the deposit surrounding the insulating pattern. Correspondingly, there are two methods to produce the seamless hollow cylinder with unidirectionally tapered perforations: electrodeposition duplication of the extruded polymer patterns (show in Figure 10) and over-growth electrodeposition from the formed insulating polymer pattern (shown in Figure 11). Except for the feeding behavior of the extruding rod, the operating steps and parameters for making the polymer mandrels (type III) showed in Figure 10 were almost the same as those for the type II mandrels. In this case, the extruding rod was kept feeding 2.3 s after the extruded melt polymer became a water-dropped shape. The type IV mandrels which are aligned with the outer surface of the stainless steel pipe (see Figure 12) were achieved just by cutting the polymer extrusions formed in any case depicted above.

**Figure 13:**
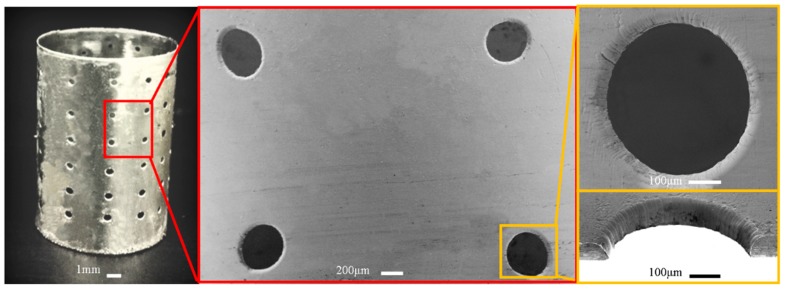
Seamless hollow cylinder with unidirectionally tapered perforations based on over-growth electrodeposition.

Based on the polymer mandrels shown in Figure 10, the electroformed seamless thin-walled cylinder (shown in Figure 11) contains unidirectionally tapered perforations formed through reversely duplicating the shape of the type III mandrels. If the electroforming was carried out based on the type IV mandrels, conic unidirectional perforations can also be obtained, but they are formed by over-growth electrodeposition process of the deposit. Compared with the perforations shown in Figure 11 which are formed via the reversely duplicating process, the perforations presented in Figure 13 are less tapered and less smooth because they are constructed through the natural over-growing process. Embosses around the orifice are apparent compared with the other type.

### 4.4. Achievement of the Seamless Hollow Cylinder with Straight-Walled Perforations

**Figure 14:**
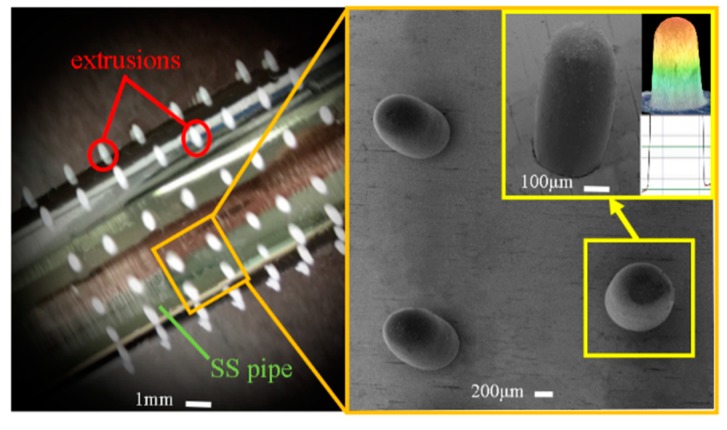
Electroforming mandrel with cylindrical dome extrusion polymer pattern.

**Figure 15:**
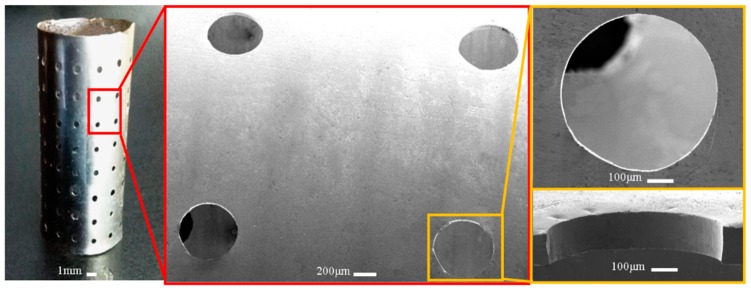
Electroformed hollow seamless cylinder with straight-walled perforations.

As shown in Figure 14, long micro-sized cylindrical pillars with a hemispherical top can be formed on the SS pipe if the following conditions and parameters for the polymer extruding operations were used: temperature of the melt polymer, 150 °C; feed rate of the extruding rod, 0.5 mm/s; feeding time, 4 s; cooling operations were the same as those described in Section 4.1. In this case, the biggest feeding rate was used and the smallest surface tension of the melt polymer was expected to be achieved so that the longest and unidiameter polymer micro-pillars could be obtained. The geometric profile of the resultant polymer pattern is highly alike the simulated one. Figure 15 shows the electroformed seamless thin-walled hollow cylinder with straight-wall perforations based on the mandrels showed in Figure 14. Obviously, the perforations are obtained in this case completely through reversely duplicating the shape of the pillar-shaped polymer patterns. Therefore, the walls of the perforations are very smooth.

## 5. Conclusions

In order to create a seamless thin-walled perforated hollow cylinder more conveniently and cost-effectively, a novel hybrid technique combining the extrusion molding process and the electroforming process was proposed. Both simulations and experiments were carried out to verify the feasibility of this proposal. Some conclusions are drawn as follows.

(1) Five types of polymer extrusion mandrels with different geometric profile were able to be achieved from the same extruding metallic pipe only by changing some extruding molding conditions and parameters, which is verified by both simulation and experimentation. The extruding molding is a very cost-effective method to create various mandrels for the electroforming process.

(2) The seamless thin-walled hollow cylinder with a great number of perforations is able to be achieved by electroforming metal on the extruding metal pipe with polymer mandrels. The orifice shape of the perforation can be changed in terms of the polymer mandrels used. Correspondingly, four kinds of perforations are created: perforation with double conic-orifices, perforation with hemispheric orifice and conic orifice, unidirectionally tapered perforation, and straight-walled perforation. The proposed hybrid technique facilitates the manufacture of seamless thin-walled hollow cylinder with various perforations.

(3) The geometric profile of the formed perforations is controlled by two formation mechanisms: over-growth electrodeposition and through-mask electrodeposition.

(4) The hybrid technique can produce thin wall cylinder component with changeable perforation in a single manner, conveniently and cost-effectively, comparing with other process. A drawback of the hybrid process is that it requires precision mode and special designed apparatus. However, this can’t overcome advantages of the technique.

## Figures and Tables

**Table 1:** Domain and boundary conditions for the simulations.

**Domain Conditions**	**Domain**	**Property and Values**
Fluid property	Ω_1_	Melt polymer
Density of melt polymer	--	0.85 g/cm^3^
Viscosity of melt polymer	--	8 N·s/m^2^
Surface tension of polymer(initial)	--	0.086 N/m
Fluid property	Ω_2_	Air
Density of air	--	1.225 kg/m^3^
Viscosity of air	--	1.789 × 10^−5^N·s/m^2^
**Boundary Conditions**	**Boundary**	**Property**
Initial interface	3	--
Inlet electrolyte	1	Laminar inflow, Φ = 1, u = u_(Inlet)_
Outlet	5, 6	P = 0 Pa
Constant(k)	--	2
wall	2, 4	u = 0
Diameter of inlet	1	0.5 mm
Diameter of outlet	5, 6	1 mm,1 mm

**Table 2:** Compositions of electrolyte and electrodeposition parameters.

Compositions of Electrolyte and Parameters (Unit)	Value
Ni(NH_2_SO_3_)_2_·4H_2_O (g·L^−1^)	360
C_12_H_25_SO_4_Na (g·L^−1^)	0.05
NiCl_2_·7H_2_O (g·L^−1^)	10
H_3_BO_3_ (g·L^−1^)	40
pH	4.5
Temperature (°C)	55
Voltage (V)	3
